# Decent work and nurses' work ability: A cross-sectional study of the mediating effects of perceived insider status and psychological well-being

**DOI:** 10.1016/j.ijnsa.2024.100283

**Published:** 2024-12-19

**Authors:** Heba Emad El-Gazar, Amira Mohammed Ali, Mona Shawer, Reham Moharam Serag, Mohamed Ali Zoromba

**Affiliations:** a Nursing Administration Department, Faculty of Nursing, Port Said University, Port Said, Egypt; b Department of Psychiatric Nursing and Mental Health, Faculty of Nursing, Alexandria University, Egypt; c Nursing Education and Advanced Practice Lead, King's College Hospital London-Jeddah, Jeddah, Saudi Arabia; d Technical Institution of Nursing, Mansoura, Egypt; e Medical Surgical Nursing Department, Faculty of Nursing, Port Said University, Port Said, Egypt; f Nursing Department, North private college of nursing, Arar, 73312, Saudi Arabia; g College of Nursing, Prince Sattam Bin Abdulaziz University, Al-Kharj, Saudi Arabia; h Psychiatric and Mental Health Nursing Department, Faculty of Nursing, Mansoura University, Mansoura, Egypt

**Keywords:** Nurses, Professional role, Psychological well-being, Work capacity evaluation, Working conditions

## Abstract

**Background:**

While the benefits of decent work—employment that respects fundamental human rights, ensures fair income, guarantees workplace security, and provides social protection for families—have recently gained scholarly attention regarding job satisfaction, psychological empowerment, and work engagement, its potential to enhance nurses' work ability—defined as the ability to carry out job responsibilities—remains unaddressed. Furthermore, a gap exists in understanding the mechanisms through which decent work influences its outcomes.

**Purpose:**

We aimed to investigate: (1) if securing decent work is associated with elevated nurses' work ability, and (2) if perceived insider status and psychological well-being mediate the association between decent work and nurses' work ability.

**Methods:**

A cross-sectional study was conducted across four public hospitals in two regions of Egypt, utilizing a self-reported survey with validated instruments, including the Decent Work Scale, Perceived Insider Status Scale, Psychological Well-being Scale, and Work Ability–Personal Radar Scale. Data analysis was performed using descriptive statistics, Pearson's correlation analyses, and structural equation modeling.

**Results:**

A total of 204 nurses were involved in this study. Decent work was positively associated with nurses' work ability, and this association was fully mediated by perceived insider status and psychological well-being.

**Conclusions:**

Cultivating decent work conditions might enable healthcare administrators to enhance nurses' sense of insider status and psychological well-being, thereby potentially boosting their work ability.

**What is already known**

• One in four nurses worldwide has low work ability, which is linked to harmful outcomes, including decreased job satisfaction, poor quality of life, and higher intentions to leave the nursing profession.

• Factors such as perceived insider status and psychological well-being are seldom considered in workforce development strategies in healthcare.

Alt-text: Unlabelled box

**What this paper adds**

• We have highlighted the benefits that decent work conditions could offer for nurses.

• Implementing policies that ensure decent work conditions for nurses has the potential to elevate their work ability.

• We have shown that perceived insider status and psychological well-being significantly mediate the relationship between work conditions and work ability, suggesting that enhancing these could improve nurses' work ability.

Alt-text: Unlabelled box

## 1 Introduction

Securing decent work practices for nurses, defined as workplace conditions that protect individuals' rights, ensure fair income, provide social protection and security, support career growth, and promote open communication (Duffy et al., 2017), has gained increased scholarly interest in the wake of its recognition as a critical element of sustainable development, as outlined in the United Nations' 2030 Agenda (World Health Organization, 2020). In particular, scholars have begun to pay close attention to the benefits of providing decent work practices for nurses. For example, researchers have indicated that nurses with access to decent work exhibit greater work engagement (BowenXue et al., 2024), psychological empowerment (Yu et al., 2023), ethical behaviors (Zoromba et al., 2024), and reduced burnout and turnover intentions (Xue et al., 2024).

While we have gained an enhanced understanding of the numerous benefits associated with nurses working in decent conditions (BowenXue et al., 2024; Yu et al., 2023; Zoromba et al., 2024), the potential for decent work to elevate nurses' work ability has, to the authors' knowledge, yet to be explored. This oversight is particularly intriguing given that a recent meta-analysis revealed that one in four nurses worldwide has low work ability (Romero-Sánchez et al., 2022)—defined by Ilmarinen et al. (2015) as the capacity to perform job duties. Therefore, we aimed to shed new light by investigating the association between decent work and the elevation of nurses' work ability. By exploring this connection, we responded to scholarly calls for more research into how decent work could yield further benefits (Ralph and Arora, 2024) and contributed to the ongoing discussion about the long-term implications that healthcare settings could gain from providing a decent work environment for nurses.

Furthermore, to develop a deeper insight into the linkage between decent work and nurses' work ability, we examined the mechanisms through which decent work could be associated with nurses' work ability. While prior researchers have indicated that decent work is related to favorable outcomes for nurses, the evidence for the underlying mechanisms mainly stems from research that tested single mediators in isolation (e.g., psychological ownership [El-Gazar et al., 2024]; work engagement [BowenXue et al., 2024]; and psychological empowerment [Yu et al., 2023]). This approach may oversimplify complex processes and fail to consider that most effects are likely driven by multiple mechanisms acting simultaneously (Hayes, 2018). Accordingly, we aimed to provide a more comprehensive analysis of the associations between decent work and nurses' work ability by examining how it operates through two distinct yet complementary mediators: perceived insider status—nurses' sense of relatedness to their organization (Kim et al., 2019)—and psychological well-being—a positive state of mental and emotional health marked by personal fulfillment and self-acceptance (Zheng et al., 2015). We selected perceived insider status and psychological well-being as dual mediators based on Social Identity Theory (Tajfel and Turner, 1979) and the Job Demands-Resources model (Bakker and Demerouti, 2007). Social Identity Theory suggests that individuals who receive favorable treatment from their organization perceive themselves as integral members (Ye et al., 2023), leading them to perform with dedication, full potential, and persistence (Kim et al., 2019; Zhao and Liu, 2020). Likewise, the Job Demands-Resources model posits that adequate resources could counterbalance the adverse effects of job demands, thereby enhancing an individual's psychological well-being (Bakker et al., 2007) and subsequently improving job performance (Lee, 2019). To this end, we aimed to investigate: (1) if securing decent work is associated with elevated nurses' work ability and (2) if perceived insider status and psychological well-being mediate the association between decent work and nurses' work ability.

## 2 Literature review and hypotheses

### 2.1 Decent work

Decent work represents the baseline attributes of employment that ensure nurses have guaranteed rights and are provided with social protection, are offered fair wages, are ensured workplace safety, are given opportunities for career advancement, and are encouraged to engage in workplace dialogue (Blustein et al., 2023; Hult et al., 2024). The concept of decent work incorporates five factors: safe conditions, adequate compensation, access to health care, complementary values, and free time and rest (Duffy et al., 2017). Access to decent work for nurses is associated with increased job satisfaction (Xue et al., 2024), enhanced teamwork ability (El-Gazar et al., 2022), better physical and mental health (Sönmez et al., 2023), and greater work immersion (Yu et al., 2023).

### 2.2 Decent work and work ability

Work ability means the capacity of nurses to meet their job requirements at a given moment and in the near future (Ilmarinen et al., 2015). Low work ability is linked to several harmful outcomes for individual nurses and healthcare settings, such as decreased job satisfaction (Tomietto et al., 2019), poor quality of life, and increased intentions to quit the nursing profession (Romero-Sánchez et al., 2022; Rongen et al., 2014).

Within the framework of the Job Demands-Resources model, the availability of job resources could initiate a motivational process that leads to greater job dedication and an enhanced capacity to meet job responsibilities (Bakker and Demerouti, 2007). Decent work, which upholds human dignity, equity, freedom, and security, is an indispensable resource for nurses (Duffy et al., 2017; Hult et al., 2024). Furthermore, Social Exchange Theory posits that workers who receive economic and socioemotional resources from their organizations are likely to feel obligated to reciprocate by fully meeting job requirements and enhancing productivity (Blau, 1964). Decent work offers financial incentives, fair wages, leadership support, job security, and work-life balance (Blustein et al., 2023), all contributing to this exchange. By applying the Job Demands-Resources model and Social Exchange Theory to the context of nursing, we argue that decent work has the potential to elevate nurses’ work ability. Consequently, we hypothesize the following.

Hypothesis 1 Decent work is positively associated with nurses' work ability.

### 2.3 Mediating role of perceived insider status

Perceived insider status means the depth at which nurses perceive themselves as integral members of their organization (Kim et al., 2019; Stamper and Masterson, 2002). Researchers have indicated that nurses who perceive themselves as insiders exhibit a higher level of presenteeism (Liu et al., 2021), enhanced self-motivation, and constructive thinking (Gong et al., 2021). Furthermore, perceived insider status is associated with increased employee commitment, organizational identification, and work engagement (Guo et al., 2022).

According to Social Identity Theory (Tajfel and Turner, 1979), nurses tend to define themselves as part of a particular organization based on the favorable treatment and the positive status they perceive within that organization (Ye et al., 2023). Nurses who work in a decent environment are well-treated by their organizations, both psychologically and materially (Blustein et al., 2023; Hult et al., 2024). Following Social Identity Theory, this appreciation, as evidenced through decent work practices, leads nurses to identify themselves as insider members. Moreover, their involvement in decision-making and the presence of open dialogue—which are recognized as hallmark features of decent work (Duffy et al., 2017)—provide them with compelling justifications to consider themselves as insiders within their organization. Expanding on this, the self-categorization theory (Turner et al., 1987) further posits that nurses who see themselves as organizational insiders are likely to embrace their work responsibilities with great dedication, full potential, and persistence (Kim et al., 2019; Zhao and Liu, 2020), thereby elevating their working ability. Consequently, we hypothesize the following.

Hypothesis 2 *Decent work is positively associated with nurses’ perceived insider status.*

Hypothesis 3 *Perceived insider status mediates the association between decent work and nurses’ work ability.*

### 2.4 Mediating role of psychological well-being

Psychological well-being refers to a positive mental and emotional state of wellness, characterized by an individual's sense of fulfillment, happiness, personal potential, life satisfaction, self-acceptance, and ability to cope with stress (Zheng et al., 2015). The Job Demands-Resources model (Bakker and Demerouti, 2007) proposes that the work environment, classified into job resources or demands, significantly affects nurses' well-being and consequently their performance (El-Gazar et al., 2023). The model also suggests that ample resources could alleviate the harmful outcomes of job demands, thereby enhancing nurses' well-being (Bakker et al., 2007; Xiao et al., 2023). Building upon the tenets of the Job Demands-Resources model, we argue that the substantial resources provided by decent work offer dual benefits. First, the acquisition of such resources could improve nurses' well-being. Second, these resources might alleviate the stress associated with the demands of the nursing profession, ultimately leading to enhanced overall well-being, including psychological well-being. Subsequent researchers on the Job Demands-Resources model have indicated that staff with higher psychological well-being perform better (Lee, 2019) and feel more competent in fulfilling their job duties (Koroglu and Ozmen, 2022), which contributes to improved work ability. Consequently, we hypothesize the following.

Hypothesis 4 *Decent work is positively associated with nurses' psychological well-being.*

Hypothesis 5 *Psychological well-being mediates the association between decent work and nurses’ work ability.*

The proposed mediation model in this study is summarized in Supplementary Material, Appendix 1.

## 3 The study

### 3.1 Aims

We aimed to investigate: (1) if securing decent work is associated with elevated nurses' work ability, and (2) if perceived insider status and psychological well-being mediate the association between decent work and nurses' work ability.

## 4 Methods

### 4.1 Study design

A cross-sectional survey was conducted and reported according to the Strengthening the Reporting of Observational Studies in Epidemiology (STROBE) guidelines.

### 4.2 Participants and setting

We surveyed bedside nurses sampled through a convenience sampling technique from four public hospitals in the regions of Port Said and Mansoura, Egypt (≤ 250-bed capacity). The target nurses were full-time registered nurses directly involved in bedside care for a minimum of 12 months and who consented to participate in the survey. Nurses were excluded if they were in leadership positions, undergoing internships, or on vacation.

We used an online calculator for structural equation modeling (SEM; Soper, 2022) to determine the required sample size. With parameters of four latent constructs, a 45-item instrument, an anticipated effect size of 0.3, a power of 90 %, and a 0.05 probability, the required sample size was 173 nurses. To account for non-responses and invalid surveys, we distributed 300 surveys.

### 4.3 Measures

The survey incorporated standardized scales, originally developed in English, to assess decent work, perceived insider status, psychological well-being, and work ability. It also collected demographic data, including participants' age, sex, marital status, education level, working unit, working shift, years in the nursing profession, and years at the current hospital. We conducted the survey in Arabic, using a back-translation procedure (Brislin, 1970). A pre-study comprising 29 nurses (excluded from the main study) recruited from two of the four hospitals under study was conducted to ensure the comprehensibility of the study survey. The participating nurses confirmed the clarity and applicability of the translated survey to our research context, and no modifications were made.

### 4.4 Decent work

We used the 15-item Decent Work Scale (Duffy et al., 2017) to evaluate the levels of decent work conditions among nurses. This scale is divided into five subscales: (a) physically and interpersonally safe working conditions, (b) access to healthcare, (c) adequate compensation, (d) hours that allow for free time and rest, and (e) organizational values that align with family and social values. Each subscale consists of three items. Participants rated items on a 7-point Likert-type scale, from 1 (strongly disagree) to 7 (strongly agree), with higher scores indicating a greater experience of decent work conditions. The Cronbach's alpha for this scale was 0.86 in the original study and 0.94 in our study.

### 4.5 Perceived insider status

We used the 6-item Perceived Insider Status Scale (Stamper and Masterson, 2002) to measure the degree to which nurses perceive themselves as insiders within their hospital. Participants responded to items on a 5-point Likert-type scale, from 1 (strongly disagree) to 5 (strongly agree), with higher scores indicating a stronger sense of perceived insider status. The Cronbach's alpha was 0.88 in both the original and current studies.

### 4.6 Psychological well-being

We used the 6-item Psychological Well-being Scale (Zheng et al., 2015) to assess the psychological well-being levels of nurses. Participants rated items on a 7-point Likert-type scale, from 1 (strongly disagree) to 7 (strongly agree), with higher scores indicating greater psychological well-being. The Cronbach's alpha was 0.82 in the original study and 0.93 in our study.

### 4.7 Work ability

We used the 18-item Work Ability–Personal Radar Scale (Ilmarinen et al., 2015) to assess the work ability levels of nurses. This scale comprises five subscales: (a) health and functional capacity (3 items); (b) competence (3 items); (c) attitudes and motivations (5 items); (d) work management (4 items); and (e) work and spare-time activities (3 items). Participants rated their agreement with each behavior on an 11-point rating scale, from 0 (cannot currently work at all) to 10 (best work), with higher scores indicating higher work ability. The Cronbach's alpha for this scale was 0.87 in the validation study (Converso et al., 2022) and 0.94 in our study.

### 4.8 Procedure

Data were collected over 3 months (January–March 2024) with permission from the chairpersons of each target hospital. Before the investigation, the first author trained four postgraduate nursing students working as staff nurses at the hospitals to act as research investigators for data collection. With the support of the nursing ward managers in each hospital, the research investigators reached eligible nurses, provided them with adequate information about the study and assured them that their responses would not be disclosed to their hospital. Nurses who agreed to participate signed informed consent forms and received a copy of the survey along with a return envelope. Completed surveys were returned directly to the research investigators in sealed envelopes within 3 days.

### 4.9 Analytical strategy

Statistical analysis was performed using SPSS Statistics 27.0 and AMOS 25.0 software. Sample demographics and clinical variables were presented using descriptive statistics. Pearson's correlation was conducted to explore the correlations between study variables. Convergent and discriminant validity were assessed for the four focal variables (i.e., decent work, perceived insider status, psychological well-being, and work ability). To establish convergent validity, factor loadings and average variance extracted (AVE) values needed to exceed 0.50, and composite reliability (CR) values needed to be above 0.70 (Hair et al., 2019). Discriminant validity was confirmed when the square root of each AVE value surpassed the correlations between variables (Fornell and Larcker, 1981) and when Heterotrait-Monotrait Ratio of Correlations values were below the recommended threshold of 0.85 (Henseler et al., 2015). Reliability was confirmed through Cronbach's alpha, with values required to exceed the minimum criterion of 0.70 (Nunnally, 1978).

The hypothesized model was examined through a two-step modeling evaluation (Anderson and Gerbing, 1988). First, Confirmatory Factor Analysis (CFA) was conducted to assess the measurement model and the validity of the study measures. Second, SEM was used to test the direct link between decent work and work ability, and its indirect link via the parallel mediating effects of perceived insider status and psychological well-being. A bias-corrected bootstrapping method with 5000 resamples and a 95 % confidence interval (CI) for the indirect effect was used. Statistical significance was reported as a two-sided *p* < 0.05.

### 4.10 Ethical consideration

The study adhered to the principles of the Helsinki Declaration and received approval from the ethics committee of the Faculty of Nursing at Port Said University, Egypt. The participating nurses were fully informed about the objectives of the research, the voluntary participation, and their ability to cease at any time. All data were collected anonymously, and identifiable details such as names and surnames were not requested.

### 4.11 Common method bias

To mitigate common method bias concerns, we implemented several procedural remedies. These included a review of the survey by relevant experts and a subsequent preliminary analysis to ensure its clarity and understandability. We also used different scale formats and prioritized the anonymity and confidentiality of participants (Podsakoff et al., 2012). After data collection, we conducted Harman's single-factor test to evaluate any potential influence of common method bias in the data. The test indicated that the maximum variance accounted for by a single factor was 37.39 %, which is below the 50 % threshold (Podsakoff and Organ, 1986), confirming that common method bias is unlikely to be a threat in this study.

## 5 Results

### 5.1 Participants' demographics

Of the 300 surveys distributed, 230 were collected, with 26 deemed invalid and removed, resulting in a final sample size of 204 participants and an effective response rate of 68 %. The participants were predominantly female (*n* = 147, 72.1 %) and married (*n* = 126, 61.8 %). The majority held a diploma in nursing (*n* = 82, 40.2 %), and their mean age was 33.91 years (SD = 7.35). Most participants worked rotating shifts (*n* = 143, 70.1 %) in medical or surgical units (*n* = 65, 31.9 %). On average, the participants had 13.75 years (SD = 6.90) of experience in the nursing profession and had worked 7.81 years (SD = 5.04) in their current hospital (Supplementary Material, Supplementary 2).

### 5.2 Measurement model

We conducted CFAs to assess the conceptual distinctiveness of our key variables. From the results, we calculated that our expected four-factor model, which included decent work, perceived insider status, psychological well-being, and work ability, yielded an acceptable fit. Furthermore, its fit indices were the best compared with those of alternative measurement models, supporting the distinctiveness of the key variables in our study (Table 1).

**Table 1:** Measurement model comparisons.

Model	χ2	df	χ2 /df	IFI	TLI	CFI	RMSEA
Hypothesized four-factor model	1401.29	929	1.51	0.93	0.93	0.93	0.050
Three-factor model (DW + PIS, PW, WA)	2139.72	937	2.29	0.83	0.82	0.83	0.080
Two-factor model (DW + PIS + PW, WA)	2413.56	939	2.57	0.83	0.79	0.78	0.088
One factor model (DW + PIS + PW + WA)	4222.12	945	4.46	0.53	0.51	0.53	0.131

Note: *N* = 204, where *N* represents the total number of participants in the study. DW, decent work; PIS, perceived insider status; PW, psychological well-being; WA, work ability.

Statistical abbreviations: χ2, chi-square; df, degrees of freedom; χ2/df, chi-square to degrees of freedom ratio; IFI, incremental fit index; TLI, Tucker-Lewis index; CFI, comparative fit index; RMSEA, root mean square error of approximation; all are measures of model fit.

### 5.3 Validity and reliability

As shown in Table 2, the loadings for all items on the scales, as well as the AVE and CR values, exceeded the minimum criteria, affirming the convergent validity of the study variables. Additionally, the square root of each AVE surpassed the correlations between variables, and the Heterotrait-Monotrait Ratio of Correlations values remained below the recommended threshold, supporting discriminant validity. The values of Cronbach's alpha exceeded the minimum threshold, confirming the reliability of the study scales.

**Table 2:** Descriptive statistics, correlations and validation.

Variable	*M* (SD)	α	Factor loadings	CR	AVE	1	2	3	4
1. Decent work	3.21 (1.16)	0.94	0.73–0.88	0.94	0.75	(0.86)	*0.49*	*0.76*	*0.59*
2. Perceived insider status	2.78 (0.99)	0.88	0.70–0.80	0.88	0.56	0.45⁎⁎	(0.75)	*0.53*	*0.68*
3. Psychological well-being	3.67 (1.22)	0.93	0.79–0.86	0.93	0.68	0.71⁎⁎	0.49⁎⁎	(0.83)	*0.66*
4. Work ability	4.29 (1.46)	0.94	0.73–0.92	0.86	0.55	0.56⁎⁎	0.62⁎⁎	0.61⁎⁎	(0.74)

Note: *N* = 204, where *N* represents the total number of participants in the study. *M*, mean; SD, standard deviation; α, Cronbach's Alpha (measure of internal consistency); AVE, average variance extracted (measure of convergent validity); CR, composite reliability (measure of convergent validity); MSV, maximum shared variance (measure of discriminant validity).

Diagonals in parentheses are the square roots of AVE. Values below the diagonal are correlation coefficients. Values in italics above the diagonal represent the Heterotrait-Monotrait Ratio of Correlations.

⁎⁎ *p* < 0.01.

### 5.4 Descriptive statistics and correlations

From the correlation analysis, we found associations among all key variables, with decent work positively correlated with perceived insider status, psychological well-being, and work ability. Additionally, perceived insider status and psychological well-being were both positively associated with work ability (Table 2).

### 5.5 Mediating effects analysis

**Fig. 1:**
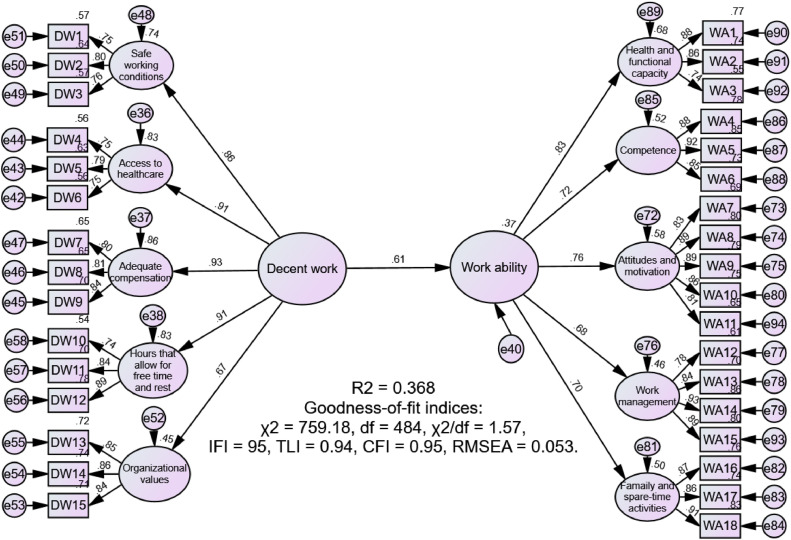
Structural model without mediators.

Note: CFI, comparative fit index; df, degrees of freedom; IFI, incremental fit index; RMSEA, root mean square error of approximation; TLI, Tucker-Lewis index.

We used SEM to analyze the research model. Initially, we tested a model linking decent work directly to nurses’ work ability without mediators. As shown in Fig. 1, decent work significantly increased nurses’ work ability, accounting for 37 % of the variance. Furthermore, the model demonstrated a good fit with the data, supporting Hypothesis 1.

**Fig. 2:**
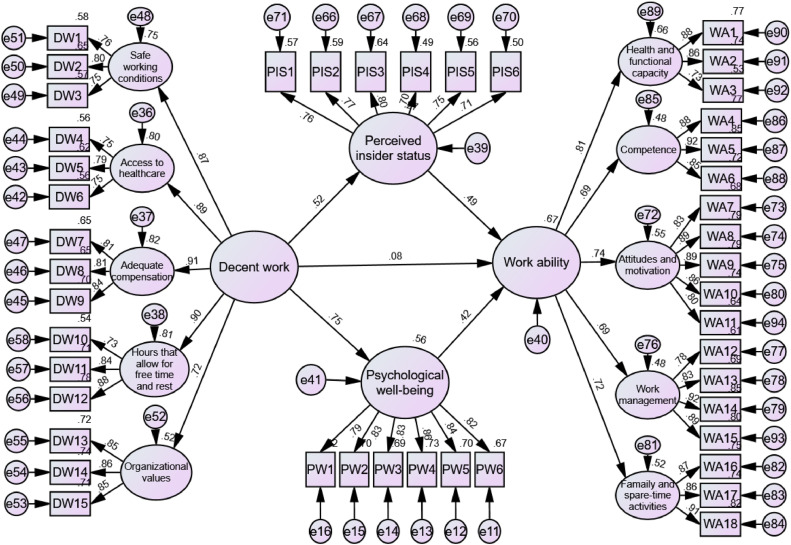
Parallel mediation model.

We then proceeded to test a model linking decent work to nurses’ work ability via the parallel mediating effects of perceived insider status and psychological well-being. As presented in Table 3 and Fig. 2, decent work significantly fostered nurses’ perceived insider status (supporting Hypothesis 2) and psychological well-being (supporting Hypothesis 4). Additionally, both perceived insider status and psychological well-being were found to positively increase nurses’ work ability.

**Table 3:** Estimates from the mediation model.

Effect	β	S.E	t	*p*	BC 95 % CI
					Lower/Upper
Direct effect					
Decent work to perceived insider status	0.52	0.09	6.05	<0.001	0.38/0.63
Perceived insider status to work ability	0.49	0.14	6.02	<0.001	0.33/0.64
Decent work to psychological well-being	0.75	0.11	8.49	<0.001	0.65/0.83
Psychological well-being to work ability	0.42	0.13	4.23	0.001	0.22/0.61
Decent work to IWI	0.08	0.18	0.75	0.454	−0.12/0.29
**Indirect effect**					
Decent work → perceived insider status → work ability	0.25			<0.001	0.27 /0.71
Decent work → psychological well-being → work ability	0.32			<0.001	0.30 /0.93
**Total effect**	0.65				0.51 /0.76

Note: *N* = 204, where *N* represents the total number of participants in the study. BC, bias-corrected; CI, confidence interval; S.E., standard error. *p* is the statistical significance level; values <0.05 are considered significant.

Standardized coefficients are reported, Bootstrap resamples = 5000.

The 5000-bootstrapping mediation analysis showed a significant indirect effect of decent work on nurses' work ability through perceived insider status (supporting Hypothesis 3) and psychological well-being (supporting Hypothesis 5). The path coefficient for the association between decent work and work ability became non-significant after incorporating the mediating variables, indicating that perceived insider status and psychological well-being fully mediated the decent work‐work ability relationship. The mediation model fit well to the data (χ²/df = 1.51, RMSEA = 0.050, CFI = 0.93, TLI = 0.93, IFI = 0.93) and explained 67 % of the variance in nurses’ work ability.

## 6 Discussion

We have offered new insights into the association between decent work and nurses' work ability. We have further explored the mediating effects of perceived insider status and psychological well-being in this association. We found that access to decent work is associated with greater work ability among nurses. Providing a decent work environment may ensure that nurses feel respected and appreciated by their workplace, and according to Social Exchange Theory, this could foster a sense of obligation to reciprocate by fulfilling all work responsibilities (Blau, 1964).

We contributed to the existing research on the potential benefits of fostering a decent work environment for nurses. While previous researchers have identified decent work as being associated with nurses' job satisfaction (Xue et al., 2024), work engagement (BowenXue et al., 2024), and psychological empowerment (Yu et al., 2023), we extended this knowledge by demonstrating that decent work may also significantly influence a critical nursing outcome: work ability. We have assessed that our findings align with those of previous researchers, suggesting that access to decent work practices may encourage nurses to work more vigorously (El-Gazar et al., 2024). Additionally, researchers have demonstrated that favorable and rewarding work-related factors are associated with enhanced nurses' work ability (Camerino et al., 2008; Chiu et al., 2007; Golubic et al., 2009; Rongen et al., 2014; Rypicz et al., 2021).

We have demonstrated that decent work is positively associated with nurses' perceived insider status. This indicates that when nurses have access to decent work, they are likely to perceive themselves as insiders within their healthcare settings. We also contributed a novel insight to the nursing literature, aligning with a prior study showing that environmental work factors may play a significant role in increasing perceived nurse insider status (Liu et al., 2021). Furthermore, our findings are consistent with those of Seren Intepeler et al. (2019), who found that positive work conditions may foster a sense of commitment among nurses to their healthcare settings. In non-nursing contexts, Sheng and Zhou (2023) found that decent work might enable employees to feel like insider members of their organizations.

Concerning the association between decent work and nurses' psychological well-being, we found that decent work conditions are significantly associated with enhanced psychological well-being among nurses. Over recent decades, numerous factors, such as mindfulness (Sulosaari et al., 2022), resilience, and low stress levels (Li and Hasson, 2020), have been identified as potential contributors to nurses' psychological well-being. We have introduced a novel possible factor—that is, working in a decent work environment. We also highlighted the potential benefits that healthcare organizations might be able to realize by adopting decent work practices. Our findings are consistent with those of previous researchers who found that decent work may enhance nurses' physical and mental health (Sönmez et al., 2023). Similarity, Hatch et al. (2018) found that environments leading to burnout had the potential to negatively impact nurses' psychological state. Furthermore, researchers in non-nursing contexts have shown that individuals with access to decent work may fulfill their fundamental needs through their employment, which ultimately could promote well-being (Ralph and Arora, 2024).

Furthermore, we found that perceived insider status and psychological well-being served as parallel mediators in the association between decent work and nurses' work ability. These are critical findings that enrich the literature by providing a novel explanation for the association between decent work and nurses' outcomes. Specifically, working in a decent environment may enhance nurses' perceived insider status and psychological well-being, which in turn could elevate their work ability. These findings support the Job Demands-Resources model, which posits that well-being acts as a mediator between work characteristics and job performance (Bakker and Demerouti, 2007). Additionally, our findings align with those of El-Gazar and Zoromba (2024), who found that a sense of belonging among nurses toward their hospitals acts as a mediator between decent work and related impacts. Moreover, these findings are in line with researchers, who showed that perceived insider status acts as a mediator between decent work and individual outcomes (Sheng and Zhou, 2023).

## 7 Practical implications

Our study has several implications for nursing practice. We have revealed that decent work is associated with elevating nurses' work ability. Therefore, nursing managers and healthcare policymakers could take action to cultivate decent work practices for nurses. To do so, they could foster a culture of fairness, develop policies that ensure mutual respect among colleagues, provide health insurance, and encourage nurses to participate in organizational dialogue and decision-making processes. Additionally, nursing managers can adopt self-scheduling, acknowledge nurses' work, offer incentives, ensure regular work breaks, and provide teaching and training opportunities for nurses.

Furthermore, nursing managers and healthcare setting administrators could find guidance in the roles played by perceived insider status and psychological well-being in the positive association between decent work and nurses' work ability. Nursing managers might consider implementing several strategies. First, they could work on making nurses perceive themselves as insiders. There are numerous strategies to achieve this, such as ensuring a workplace free of incivility (Guo et al., 2022) and eliminating harmful leadership behaviors (Ye et al., 2023). Second, nursing managers may benefit from paying attention to enhancing nurses' psychological well-being. This can be achieved by being more supportive, providing feedback (Zhao and Liu, 2020), and fostering self-esteem and optimism, as well as combating job discrimination (Lee, 2019).

## 8 Limitations and future research

The research had several limitations. First, because of the cross-sectional design, the potential for reverse causality cannot be ruled out. Future studies can validate our findings by employing different research designs, such as experimental or longitudinal analyses. Second, although the data were collected from two different regions in Egypt, the study is still limited to a national cultural context. We call for future research to capture data from various contexts to enhance the generalizability of the study findings. Third, the study variables were measured using individual self-reports, which can raise concerns about common method bias. Despite the results indicating that common method bias in this study was not a concern, we recommend using multisource methods, such as observations, in future research. Finally, we examined the benefits of decent work by focusing only on elevating nurses' ability. While our findings contribute to the existing literature on the benefits of decent work, we recommend that future research investigate the predictive effects of decent work on other possible outcomes, such as a strength mindset, commitment, and voice behaviors. Researchers on these topics could add significant value to nursing literature and management practices.

## 9 Conclusions

We have advanced knowledge on the association between decent work and nurses' work ability, including the parallel mediating effects of perceived insider status and psychological well-being. We have demonstrated that decent work was positively associated with nurses' work ability. Furthermore, we have shown that perceived insider status and psychological well-being mediated the association between decent work and nurses' work ability.

## Funding statement

This research received no specific grant from any funding agency in the public, commercial, or not-for-profit sectors.

## CRediT authorship contribution statement

**Heba Emad El-Gazar:** Writing – original draft, Conceptualization. **Amira Mohammed Ali:** Writing – original draft, Methodology. **Mona Shawer:** Methodology. **Reham Moharam Serag:** Writing – original draft. **Mohamed Ali Zoromba:** Writing – original draft, Methodology, Conceptualization.

## Declaration of competing interest

The authors declare that they have no known competing financial interests or personal relationships that could have appeared to influence the work reported in this paper.

## Data Availability

The data that support the findings of this study are available from the corresponding author upon reasonable request.

## Appendix Supplementary materials

Image, application 1

## Footnotes

Supplementary material associated with this article can be found, in the online version, at doi:10.1016/j.ijnsa.2024.100283.
