# Supplementary material for: Decent work and nurses' work ability: A cross-sectional study of the mediating effects of perceived insider status and psychological well-being
Source: Int J Nurs Stud Adv. 2024 Dec 19;8:100283. doi: 10.1016/j.ijnsa.2024.100283 (PMC11733275; doi:10.1016/j.ijnsa.2024.100283)
Supplement: Supplementary file 1 [file mmc1.docx]

Decent

work

Work

ability

Psychological well-being

Perceived insider status

**Appendix 1**. The Proposed Mediation Model

| **Appendix 2.**  **Characteristics of the sample** | | | | | |
| --- | --- | --- | --- | --- | --- |
| Characteristic | Category | *n* | Percent | Mean (SD) | Range |
| Age (years) | ≤30 | 63 | 30.9 | 33.91 (7.35) | 19-53 |
|  | >30 | 141 | 69.1 |  |  |
| Sex | Male | 57 | 27.9 |  |  |
|  | Female | 147 | 72.1 |  |  |
| Marital status | Never married | 61 | 29.9 |  |  |
|  | Married | 126 | 61.8 |  |  |
|  | Ever married | 17 | 8.3 |  |  |
| Education | Diploma | 82 | 40.2 |  |  |
|  | Associate | 49 | 24.0 |  |  |
|  | Bachelor | 59 | 28.9 |  |  |
|  | Postgraduate | 14 | 6.9 |  |  |
| Working unit | Medical/surgical | 65 | 31.9 |  |  |
|  | Critical care | 61 | 29.9 |  |  |
|  | Oncology/ Emergency | 48 | 23.5 |  |  |
|  | Others^†^ | 30 | 14.7 |  |  |
| Years as a nurse | ≤10 | 68 | 33.3 | 13.75 (6.90) | 2-30 |
|  | >10 | 136 | 66.7 |  |  |
| Years in the current hospital | ≤5 | 87 | 42.6 | 7.81 (5.04) | 1-28 |
|  | >5 | 117 | 57.4 |  |  |
| Working shift | Rotating shift | 143 | 70.1 |  |  |
|  | Fixed day | 25 | 12.31 |  |  |
|  | Fixed night | 36 | 7.6 |  |  |
| Note: *N* = 204, where N represents the total number of participants in the study. *M* = mean; *n* = number of participants; SD = standard deviation. ^†^ Included operation theaters, pediatric departments, orthopedic departments, and maternity units. | | | | | |
